# Systematic and MRI-Cognitive Targeted Transperineal Prostate Biopsy Accuracy in Detecting Clinically Significant Prostate Cancer after Previous Negative Biopsy and Persisting Suspicion of Malignancy

**DOI:** 10.3390/medicina57010057

**Published:** 2021-01-10

**Authors:** Alvydas Vėželis, Gediminas Platkevičius, Marius Kinčius, Liutauras Gumbys, Ieva Naruševičiūtė, Rūta Briedienė, Donatas Petroška, Albertas Ulys, Feliksas Jankevičius

**Affiliations:** 1 Department of Oncourology, National Cancer Institute, 08406 Vilnius, Lithuania; alvydas.vezelis@nvi.lt (A.V.); marius.kincius@nvi.lt (M.K.); albertas.ulys@nvi.lt (A.U.); 2 Institute of Clinical Medicine, Faculty of Medicine, Vilnius University, 03101 Vilnius, Lithuania; feliksas.jankevicius@santa.lt; 3 Department of Radiology, Nuclear Medicine and Physics of Medicine, Center for Radiology and Nuclear Medicine, Vilnius University Hospital Santaros Klinikos, 08661 Vilnius, Lithuania; liutauras.gumbys@santa.lt; 4 Department of Diagnostic and Interventional Radiology, National Cancer Institute, 08660 Vilnius, Lithuania; ieva.naruseviciute@nvi.lt (I.N.); ruta.briediene@nvi.lt (R.B.); 5 National Center of Pathology, Affiliate of Vilnius University Hospital Santaros Klinikos, 08406 Vilnius, Lithuania; donatas.petroska@vpc.lt

**Keywords:** cognitive targeted prostate biopsy, prostate cancer, multi-parametric magnetic resonance imaging, transperineal prostate biopsy

## Abstract

*Background and objectives:* Overdiagnosis, overtreatment, and the need for repeated procedures caused by transrectal ultrasound guided prostate biopsies and their related complications places a heavy burden on healthcare systems. This was a prospective cohort validating study to access the clinical accuracy of systematic and MRI-cognitive targeted transperineal prostate biopsies in detecting clinically significant prostate cancer after a previous negative biopsy and persistent suspicion of malignancy. The primary goal was to assess the ability of multiparametric magnetic resonance imaging (mpMRI) to detect clinically significant prostate cancer with an additional goal to assess the diagnostic value of systematic and MRI-cognitive transperineal biopsies. *Materials and Methods:* In total, 200 patients were enrolled who had rising serum prostate specific antigen (PSA) levels for at least 4 months after a previous negative transrectal ultrasound (TRUS) biopsy. All eligible men underwent 1.5T prostate mpMRI, reported using the Prostate Imaging Reporting and Data System version 2 (PI-RADS v2), followed by a 20-region transperineal prostate systematic biopsy and additional targeted biopsies. *Results:* Systematic 20-core transperineal prostate biopsies (TPBs) were performed for 38 (19%) patients. Systemic 20-core TPB with additional cognitive targeted biopsies were performed for 162 (81%) patients. Clinically significant prostate cancer (csPC) was detected for 31 (15.5%) patients, of which 20 (64.5%) cases of csPC were detected by systematic biopsy, eight (25.8%) cases were detected by targeted biopsy, and three (9.7%) both by systematic and targeted biopsies. *Conclusions:* Cognitive mpMRI guided transperineal target biopsies increase the detection rate of clinically significant prostate cancer after a previously negative biopsy. However, in a repeat prostate biopsy setting, we recommend applying a cognitive targeted biopsy with the addition of a systematic biopsy.

## 1. Introduction

Prostate cancer (PC) is the second most common cancer and the fifth most common cause of cancer-related death in men globally [1]. PC has a complex diagnostic pathway, and men with an increased risk or clinical suspicion are identified using a blood serum marker, prostate specific antigen (PSA), in combination with radiological tests and pathological results [2].

In cases of clinical suspicion of prostate cancer, patients undergo a systematic transrectal ultrasound-guided biopsy (TRUS biopsy) as the next step, during which 8 to 12 core samples are taken. However, TRUS biopsies have begun to show their own limitations, such as low accuracy of detecting clinically significant prostate cancer (csPC), high rates of false positive results, and Gleason score underestimations as high as 47.8% [3].

Up to one million TRUS biopsies are performed yearly in the United States [4], and about 80,000–90,000 in the United Kingdom [5]. The overdiagnosis and overtreatment caused by this test and its related complications places a heavy burden on healthcare systems [6], in addition to creating the need for repeat biopsies in cases where PC suspicion persists [7].

The number of transperineal template prostate mapping biopsies (TTPM biopsies) has risen recently [8], which, although costlier, have a lower rate of infectious complications [9]. These biopsies can be performed either by the “fan technique” (FT) or the co-axial technique. The FT is performed by sampling the prostate through a single access of each prostatic lobe, changing the needle angle to reach the targeted area. The co-axial technique is performed by puncturing the skin and other tissues for each single core taken [10]. TTPM biopsies allow for systemic prostate sampling every 5 mm (using a standard brachytherapy grid), thus vastly improving diagnostic accuracy. As shown by Barzell et al., TTPM biopsies in a repeat biopsy setting were superior to a TRUS approach, with up to 76–80% csPC cases underdiagnosed if only TRUS biopsies were performed [11].

Another advantage of the TTPM biopsy is the possibility to sample the apical/anterior prostate zones, which are usually difficult to reach during TRUS biopsies and prone to undersampling in a repeat biopsy setting [12,13]. TTPM biopsies clearly have their own shortcomings—there is a need for general anesthesia in most centers and acute urinary retention is more frequent, occurring in almost 17% of patients [8]. 

Considering the drawbacks of these invasive tests and risk of associated complications, especially in patients with prior negative TRUS biopsies, a better test is required for men who indeed need a repeat biopsy.

Multiparametric magnetic resonance imaging (mpMRI) is widely reported as a valuable tool in the PC diagnostic pathway and its use is increasing in clinical practice as a test with moderately high sensitivity and varied but high negative prognostic value in detecting csPC [14,15,16]. Moreover, suspicious lesions on mpMRI may help to perform targeted biopsies, and further improve the accuracy of prostate cancer detection and reduce the number of unnecessary random biopsies [17,18,19]. There are three main approaches to perform mpMRI-targeted prostate biopsy. One is with the help of specific devices of algorithm-based fusion software [20]. Second is the so-called direct in-bore magnetic-resonance-guided biopsy when the biopsy is performed with live MRI imaging after each sample. It can also be performed cognitively seeing the location of the suspicious lesion on the magnetic resonance images when using a real-time ultrasound device without any additional software [21,22]. Direct in-bore MRI-guided biopsies and fusion software-guided biopsies require additional expensive hardware and software that may be not available in many urology centers. Furthermore, Pahwa and colleagues evaluated the cost-effectiveness of these three different MRI-guided biopsy strategies and came to the conclusion that, to date, cognitively guided MR biopsies are the most cost-effective approach [23].

This was a prospective cohort validating study with the primary goal to access the clinical accuracy of systematic and MRI cognitive targeted transperineal prostate biopsies in detecting clinically significant prostate cancer after a previous negative biopsy and persisting suspicion of malignancy.

## 2. Materials and Methods

### 2.1. Patient Population

Patients with persistent suspicion of PC due to positive digital rectal examination and a rising prostate specific antigen no less than four months after a prior negative 8 to 12 core systemic transrectal prostate biopsy were evaluated in our department between April 2016 and September 2018.

Regional Biomedical Research Ethics Committee (Document No. 15820-16-842-348) approved the study.

Men were eligible if they were under 75 years of age and gave an informed consent. Patients that met the inclusion criteria were included in the study consecutively. Ineligibility criteria were refusal to participate in the study, prior prostate surgical interventions (transurethral prostatectomy, adenomectomy, suprapubic fistulae), acute urinary tract infections, bacterial prostatitis, other malignancy excluding skin cancers if less than 5 years have passed after surgery, renal function impairment and metal implants or foreign bodies precluding an MRI examination.

### 2.2. Imaging

All eligible patients underwent mpMRI using a 1.5 T (Philips Achieva) machine with a pelvic coil. The protocol included axial, sagittal, and coronal T2-weighted (T2W) imaging (slice thickness 4 mm), and multiple b-value (b = 0, 500, 1000) diffusion-weighted imaging (DWI) with a high b value apparent diffusion coefficient (ADC) map and dynamic contrast enhancement (DCE) e-THRIVE sequence with gadolinium (temporal resolution 7 s). mpMRIs were reported by a single experienced urologic radiologist using PI-RADS v2.

**Figure 1:**
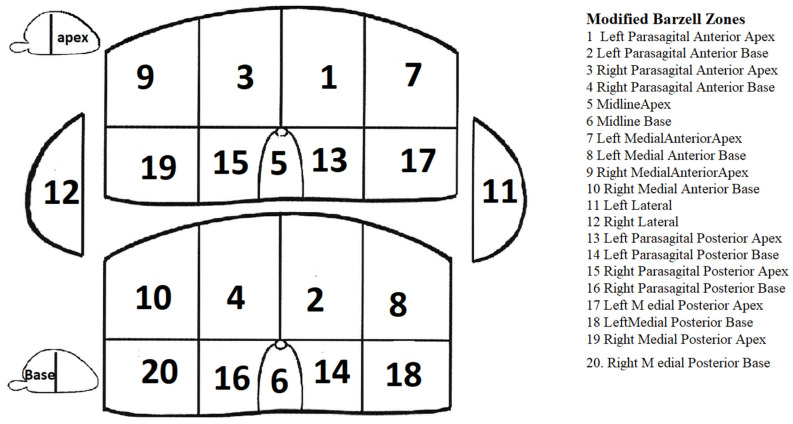
Modified 20-zone prostate scheme for the transperineal template prostate mapping (TTPM) biopsy, according to Barzell [24].

**Figure 2:**
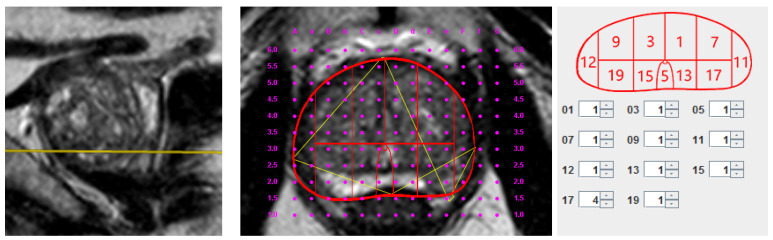
Multiparametric magnetic resonance imaging (mpMRI) image adjusted to brachytherapy grid coordinates with a positive Prostate Imaging Reporting and Data System (PI-RADS) 4 lesion. PI-RADS v2 score of 4 in zone 17 of the modified Barzell scheme, corresponding to coordinate E2.0 in the brachytherapy grid (urethra in coordinate D2.5).

The identified lesions were mapped on a 20-zone schema (Figure 1), superimposed on an axial T2W image together with a standard brachytherapy grid used in transperineal biopsies. Lesion PI-RADS v2 score, its occupied prostate zone, and brachytherapy grid coordinates were indicated (Figure 2). In addition, prostate volume was calculated using the standard ellipsoid volume formula (length × width × height × 0.52) and was used in calculating prostate specific antigen density. Prostate lesions were determined as clinically significant if the PI-RADS v2 score ≥ 3 was assigned.

### 2.3. Biopsy

Two weeks after the mpMRI examination, the patients underwent a 20-zone transperineal prostate biopsy using a brachytherapy grid in lithotomy position, performed by a single urologist who had the mpMRI report. Procedures were carried out under general anesthesia and with an antibiotic prophylaxis. Each zone was sampled cognitively once. Each mpMRI PI-RADS ≥3 lesion ≥5 mm was cognitively biopsied. One additional biopsy per lesion was performed. No complications were registered after TTPM.

### 2.4. Histopathology

Biopsy cores were analyzed by a single experienced uropathologist who was blinded to the mpMRI results. The changes were evaluated using the Gleason grading system in concordance with the 2014 International Society of Urological Pathology (ISUP) guidelines [25].

The pathology results were divided into three histological groups—clinically significant cancer, clinically insignificant cancer, and benign findings. There is a plethora of clinically significant prostate cancer definitions found in the literature, using the dominant Gleason pattern or the volume of the tumor, but to date there is no universally accepted definition [26]. Clinically significant prostate cancer was defined as suggested by the University College London collective: Gleason grade ≥ 3 + 4 and/or cancer core length (CCL) ≥ 6 mm [27].

### 2.5. Statistics

Descriptive statistics were used to summarize patient characteristics (age, PSA, prostate volume). Logistic regression analysis was used to evaluate possible prognostic factors (prostate volume, PSA, prostate specific antigen density (PSAD), mpMRI) for csPC. The Youden Index was calculated and the corresponding cut-off value for the highest Youden Index was considered as the optimal cut-off value predicting csPC. *p* < 0.05 was considered to indicate statistical significance. The statistical analysis was performed using SPSS (ver. 24; SPSS Inc., Chicago, IL, USA) and SAS (ver. 9.2; Cary, NC, USA).

## 3. Results

In total, 200 men were included. Detailed patient characteristics are shown in Table 1. At mpMRI, 38 (19%) patients were in PI-RADS v2 categories 1–2, 27 (13.5%) were scored as PI-RADS v2 3, 102 (51%) as PI-RADS v2 4 and 33 (16.5%) as PI-RADS v2 5. A systematic 20-core TTPM biopsy was performed in 38 patients with PI-RADS v2 results < 3. A systematic 20-core TTPM biopsy with additional cognitive targeted biopsy (one per lesion) was performed for 162 patients with PI-RADS results 3 or higher.

In general, 102 (51%) men had prostate adenocarcinoma of which 78 (39%) were Gleason 6 (3 + 3), 18 (9%) Gleason 7 (3 + 4), and 6 (3%) patients with Gleason 7 (4 + 3) patterns. Using the mentioned histological criteria, there were 31 (15.5%) cases of csPC. In the group of systematic biopsies only, three patients had csPC biopsy results (7.9%). In the group of cognitive targeted biopsies together with systematic biopsy, 17 cases of csPC were detected by systematic biopsy, eight cases were detected by targeted biopsy, and three cases were detected both by systematic and targeted biopsies. Detailed characteristics of biopsy results are listed in Table 2.

Topographical analysis of cognitive targeted biopsies showed that of the 11 newly found csPC lesions in eight patients, four (36%) were in the anterior base zone, 3 (27%) were in the anterior apex zone, two (18%) were in the lateral zone, and one (9%) of each were in the posterior base and posterior apex zones of the prostate.

**Figure 3:**
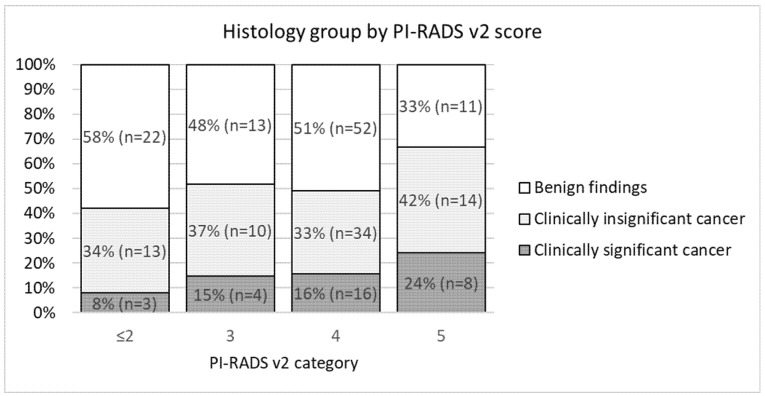
Stacked column chart demonstrating the histological outcome on transperineal template prostate mapping biopsies for PI-RADS v2 categories.

Figure 3 demonstrates the distribution of TTPM biopsy-determined histological groups (clinically significant cancer, clinically insignificant cancer, and benign) in different PI-RADS v2 categories. csPC detection increased from 8% in PI-RADS categories 1–2 to 24% in the PI-RADS 5 category.

Univariate logistic regression revealed that PSA, PSAD, and prostate volume were statistically significant factors for csPC appearance: odds ratio (OR) (95% CI, *p* value) were 1.13 (1.02–1.27, *p* = 0.021), 15.68 (2.05–119.64, *p* < 0.008), and 0.96 (0.94–0.98, *p* < 0.001), respectively.

The cut-off value of PSAD to diagnose csPC was calculated as 0.15.

When using a PI-RADS v2 score of ≥3 as a positive test result to detect clinically significant prostate cancer, 162 (81%) had a positive prostate mpMRI with sensitivity of 90.3% (95% CI: 74.3–97.9%), specificity of 20.7% (95% CI: 14.9–27.6%), negative predictive value (NPV) 92.1% (95% CI: 79.3–97.3%), and positive predictive value (PPV) 17.3% (95% CI: 15.4–19.4%) (Table 3). Accuracy assessed by area under receiver operating characteristic (AUROC) curve was 0.555 (95% CI: 0.483–0.625).

**Figure 4:**
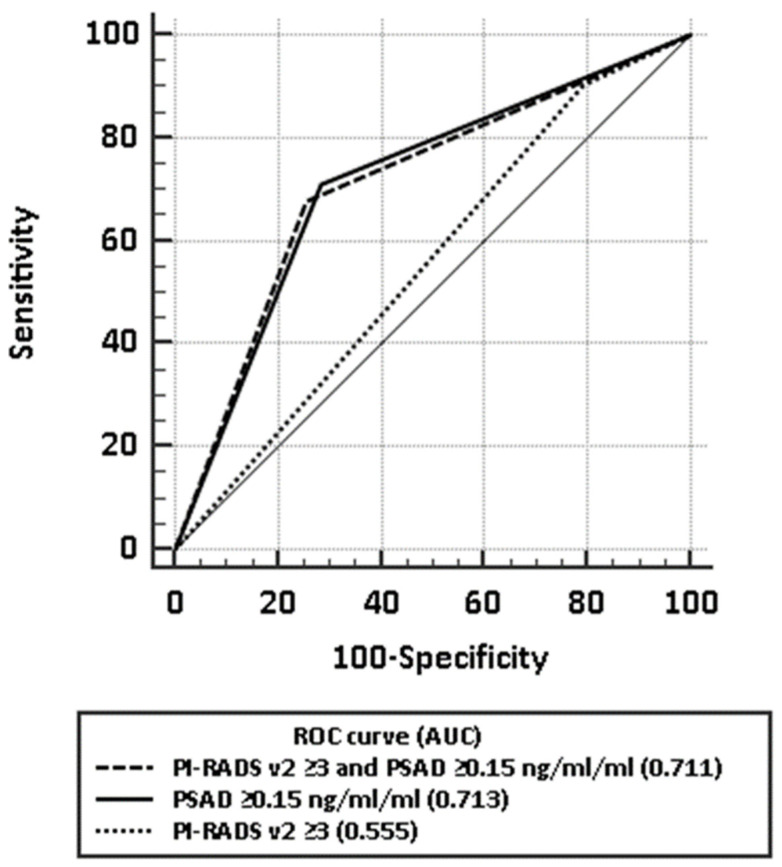
Comparison of receiver operating characteristic (ROC) curves for mpMRI alone, PSAD alone, and a model of mpMRI combined with PSAD in detecting clinically significant prostate cancer.

Using a combined model of mpMRI and PSAD (PI-RADS v2 ≥ 3 and PSAD ≥ 0.15 ng/mL/mL) to detect clinically significant prostate cancer, the sensitivity, specificity, NPV, and PPV were 67.7% (95% CI: 48.6–83.3%), 74.6% (95% CI: 67.3–80.9%), 92.6% (95% CI: 88.2–95.5%), and 32.8% (95% CI: 25.5–41%), respectively, with AUROC of 0.711 (95% CI: 0.643–0.773) (Table 3). Comparison of ROC curves using mpMRI alone, PSAD alone, and a model of mpMRI combined with PSAD are demonstrated in Figure 4. No significant difference was found between AUC of PSAD alone and the combined model.

## 4. Discussion

According to the latest guidelines, fusion targeted prostate biopsies are highly recommended as the first-choice diagnostic tool in a repeated prostate biopsy setting. However, the fusion biopsy software tool is still not widely available. In this case, we examined the performance of cognitive MRI-targeted prostate biopsies. We found that cognitive MRI-targeted transperineal biopsies significantly improved the detection rate of clinically significant prostate cancer, in general, by 26% of systematically undetected csPC cases. However, only 6.8% of all MRI positive patients showed a targeted biopsy positive with csPC. This could be associated with the poor specificity.

Due to poor specificity, the diagnostic accuracy of mpMRI was low, as indicated by AUROC of 0.56 (significantly lower than the widely accepted 0.8 threshold), and a high rate of false positives confirms that a positive mpMRI cannot substitute for a systematic TTPM biopsy.

In addition to PSA, PSAD is commonly used as a more cancer-specific derivative, yet in studies the cut-off for csPC varies from 0.08 to 0.30 ng/mL/mL [28,29], with 0.15 and 0.2 ng/mL/mL used most commonly [30].

Our results suggest that using a combined mpMRI–PSAD model (cut-off of PI-RADS v2 ≥ 3 and PSAD ≥ 0.15 ng/mL/mL) could potentially increase the specificity at the cost of sensitivity, allowing for more than three times as many patients to avoid a repeat a biopsy compared to using mpMRI only with a similar percentage of missed csPC. This is in concordance to other published data. Zalesky et al. performed a study of 397 patients and found that a combination of mpMRI and PSAD might reduce the number of biopsies performed by 21.5% with the cost of missing <4% of csPCa [14]. Furthermore, Washino et al. analyzed data of 288 patients and found that the same combination was associated with detection rates for csPCa of 76–97% and could reduce the number of unnecessary biopsies [31].

According to our data, if mpMRI is scored PI-RADS v2 ≤ 2 and a repeat biopsy is deemed unnecessary, csPC would be underdiagnosed in less than 1 in 10 men, in keeping with a similar to the “PICTURE study” published by Simmons et al. in 2017 [32]. In our cohort, there were three such cases: two 6 mm Gleason 3 + 3 pattern tumors and one 8 mm Gleason 3 + 3 case. These cases were classified as clinically significant by the csPC definition that we used for our study and suggested by the University College London collective: Gleason grade ≥3 + 4 and/or cancer core length (CCL) ≥ 6 mm [27].

One of the main limitations of our study may be sampling a single core from each zone in the 20-zone scheme of a systematic biopsy, disregarding the prostatic volume. Although the optimal number of biopsy cores remains in dispute [33], other studies using transperineal template prostate biopsies report taking 23–53 cores [31,34,35,36]—20 cores may not be enough to sample the whole craniocaudal length of larger glands and adjusting the sample number to prostatic volume could potentially reduce the chance of sampling error and false negative results. We have also performed cognitive targeted biopsies in a scheme—one sample per lesion, disregarding the lesion volume, when it was in diameter of at least 5 mm, which could have also limited the accuracy of targeted biopsies.

Additionally, we have no statistical comparability of radiological performance in our study because we have registered a high number of false positive PI-RADS 3 and 4 scores—48% of PI-RADS 3 cases were false positive and 51 % of PI-RADS 4.

The current European Association of Urology (EAU) guidelines on prostate cancer recommend performing a targeted biopsy only on patients with a prior negative biopsy and a clinical suspicion of prostate cancer [37]. However, the study that this recommendation was supported by showed the higher added value for the MRI-targeted biopsy than for the systematic biopsy (9.6% (7.7–11.8) vs 2.3% (1.2–4.5)) when comparing all kinds of MRI-targeted techniques (software, cognitive, in-bore) [38]. Our study showed that performing a cognitive MRI-targeted prostate biopsy without performing a systematic biopsy leaves a high number of csPC cases undetected.

## 5. Conclusions

Cognitive mpMRI-guided transperineal target biopsies after a previously negative prostate biopsy increased the detection rate of clinically significant prostate cancer in our study when combined with a systematic TTPM biopsy. However, we do not recommend applying a cognitive targeted biopsy only, without performing a systematic biopsy in a repeat biopsy setting.

## Figures and Tables

**Table 1:** Patient demographic data, PI-RADS v2, and Gleason pattern characteristics, clinically significant prostate cancer (csPC) at TTPM biopsy.

**Patient Characteristics**	***N***
Age, mean (SD), years	62 (5.9)
PSA at first biopsy, median (IQR), ng/mL	5.6 (4.8–6.17)
PSA at TTPM biopsy, median (IQR), ng/mL	7.6 (5.6–10.9)
Prostate volume at TTPM biopsy, median (IQR), mL	62 (46.9–87.5)
PSAD at TTPM biopsy, median (IQR), ng/mL/mL	0.121 (0.084–0.187)
Days between the first TRUS biopsy and repeated TTPM biopsy, mean (IQR), days	1081 (708–2064)
**PI-RADS v2 category**	***N* (%)**
PI-RADS 1–2, *n* (%)	38 (19%)
PI-RADS 3, *n* (%)	27 (13.5%)
PI-RADS 4, *n* (%)	102 (51%)
PI-RADS 5, *n* (%)	33 (16.5%)
**Gleason pattern**	***N* (%)**
Benign, *n* (%)	98 (49%)
Gleason 3 + 3, *n* (%)	78 (39%)
Gleason 3 + 4, *n* (%)	18 (9%)
Gleason 4 + 3, *n* (%)	6 (3%)
**Clinically significant cancer at TTPM biopsy**, *n* (%)	31 (15.5%)

Abbreviations: SD—standard deviation, IQR—interquartile range, PSAD—prostate specific antigen density TTPM—transperineal prostate mapping.

**Table 2:** Detailed characteristics of prostate biopsy results. Percentage values are calculated of total targeted biopsies performed.

Biopsy Type on Which PC Was Found for Each Patient	PI-RADS v.2	(PI-RADS < 3)		(PI-RADS ≥ 3)		Total	
		**PC**	**csPC**	**PC**	**csPC**	**PC**	**csPC**
**Systematic**, *n* (%)		16	3	38	17 (10.5%)	54	20
**Targeted**, *n* (%)	3	-	-	2	1 (0.6%)	9	8
	4	-	-	3	4 (2.7%)		
	5	-	-	4	3 (1.8%)		
**Systematic + Targeted**, *n* (%)	3	-	-	5	-	39	3
	4	-	-	22	2 (1.2%)		
	5	-	-	12	1 (0.6%)		
**No Cancer**, *n*		22		76		98	

Abbreviations: PC—Prostate cancer; csPC—clinically significant prostate cancer; Systematic—number of cases, detected by systematic TTPM biopsy. Targeted—number of cases, detected by targeted TTPM biopsy. Systematic + targeted—number of cases that have been detected both by systematic and by targeted biopsies.

**Table 3:** Detailed characteristics of prostate biopsy results. Percentage values are calculated of total targeted biopsies performed.

Parameter	Beta Coefficient	SE	Walds Statistics	Odds Ratio	Odds Ratio 95% CI	*p* Value
Baseline PSA	0.13	0.06	5.31	1.14	1.02–1.27	0.021
PSAD	2.75	1.04	7.04	15.68	2.05–119.64	0.008
Prostate volume	–0.04	0.01	15.18	0.96	0.94–0.98	<0.001
PiRADS ≥ 3	0.442	0.668	0.437	1.56	0.42–5.76	0.509
PSA density > 0.15	1.759	0.438	16.162	5.81	2.46–13.69	<0.001
PiRADS ≥ 3 and PSA density > 0.15	1.817	0.423	18.463	6.15	2.69–14.09	<0.001

Abbreviations: PC—Prostate cancer; csPC—clinically significant prostate cancer; PSAD—prostate specific antigen density; SE—standard error.

## Data Availability

Data sharing is not applicable to this article.
